# Surgical correction of a ventricular septal defect in a child with spinal muscular atrophy type 2 treated with nusinersen sodium: a case report

**DOI:** 10.1186/s13019-023-02170-z

**Published:** 2023-02-09

**Authors:** Mehmet Biçer, Şima Kozan, Figen Öztürk, Ayfer Arduç Akçay

**Affiliations:** 1 Department of Pediatric Cardiovascular Surgery, Koç University Hospital, Istanbul, Turkey; 2 School of Medicine, Koç University, Koç University Hospital, Zeytinburnu, Istanbul, Turkey; 3 Department of Anesthesia and Reanimation, Erzurum Regional Training and Research Hospital, Erzurum, Turkey; 4 Department of Pediatric Neurology, Koç University Hospital, Istanbul, Turkey

**Keywords:** Spinal muscular atrophy, Ventricular septal defect, Pulmonary banding, Neuromuscular disorders, Congenital heart defects

## Abstract

**Introduction:**

Spinal muscular atrophy (SMA) is a severe, inherited neuromuscular disorder characterized by progressive muscle weakness and atrophy. Cardiac pathology co-existence is reported more frequently in the severely affected patient groups. Structural heart anomalies, mainly septal, and outflow tract defects are commonly observed pathologies.

**Case presentation:**

We herein report the case of a 23 days-old female patient with the diagnosis of spinal muscular atrophy type 2 complicated with structural heart defects. Successful pulmonary banding, and at the age of 17 months, subsequent surgical atrial and ventricular septal defect closure were performed on our patient who was under treatment of Nusinersen Sodium. Post-operative recovery was uncomplicated. Cardiac assessments were normal, and the patient was neurologically improving in her recent follow-up.

**Conclusion:**

In the literature, there are no reported cases of successful surgical repair of heart defects in spinal muscular atrophy patients. These patients can be perceived as risky surgical candidates with suboptimal postoperative recovery given the unfavorable disease prognosis of SMA in untreated patients. We report our promising experience with a SMA type 2 patient undergoing a disease-modifying medical treatment. The SMA patients under treatment may be potential candidates for successful surgical cardiac correction given their overall improved prognosis.

**Supplementary Information:**

The online version contains supplementary material available at 10.1186/s13019-023-02170-z.

## Background

Spinal muscular atrophy is estimated to affect 1 in 11,000 live births and is one of the leading causes of infantile mortality [1, 2]. The lack of SMN-1 gene expression is the main cause of this disease [1–4]. Reports of cardiac pathology co-existence with SMA are more frequent among the severely affected patients [4]. SMA has been historically associated with a poor disease prognosis, but with the introduction of disease-modifying medical treatments, spinal muscular atrophy is now associated with a better survival overall [1]. Also, the considerably enhanced capacity for surgical correction of the heart has transformed the perspective, and surgical outcomes for many patients with hereditary syndromes are now much improved than they were previously [5]. We present a promising case of a successful two-step total cardiac surgical correction performed on a patient with SMA type 2.

## Case report

The patient was a 23 days-old female referred from the neonatal ICU with a spinal muscular atrophy type 2 diagnosis received in-utero associated with patent ductus arteriosus, ventricular, and atrial septal defects. She had an older sibling diagnosed with SMA and an amniocentesis was requested by the family. At admission, she suffered from dyspnea and poor feeding. Survival motor neuron-1 (SMN-1) gene exon 7 and 8 homozygous deletion and two copies of SMN-2 gene were detected in genetic testing. Regarding the surgical correction plan, given the post-operative morbidity potential in this patient as well as the uncertainty of the future access to disease-modifying treatments, a palliative procedure was decided upon. At the age of 23-days and a weight of 3.7 kg, we performed pulmonary banding and patent ductus arteriosus ligation under anesthesia with 4 mg Thiopental, 4mcg Fentanyl, and muscle blockage with 1.5 mg Rocuronium. The patient was extubated 16 h after the operation with a stable hemodynamic status. The postoperative echocardiographic evaluation revealed a 55 mmHg gradient on the pulmonary band and no residual flow from the patent ductus arteriosus (Additional file 1: Fig. S1). The patient was discharged on postoperative 10th day. During this course, the patient was neurologically asymptomatic other than absent deep tendon reflexes and had not yet received the first dose of her treatment with Nusinersen: an antisense oligonucleotide that increases the synthesis of full-length survival motor neuron protein that is lacking in spinal muscular atrophy patients [1]. At 12 months of age, before her first dose, the patient received a score of 3 on the Hammersmith Functional Motor Scale Expanded (HFMSE) [6]. She received her first dose of treatment at the age of 14 months.

The second admission of the patient was at age 17 months. The pulmonary band gradient was 78 mmHg, and pulse oxygen saturation was 93. No neurologic deterioration was noted, patient was able to hold her head, sit unsupported and the weighed 8.2 kg. We performed atrial and ventricular septal defect closure and pulmonary artery reconstruction. Neuromuscular blockade was established with Rocuronium 4 mg; sedation was applied with Pentothal 25 mg, Dormicum 0.5 mg, Fentanyl 16 mg, and maintained with Precedex 0.1 mg/kg/h and Sevoflurane 0.6–0.7 MAC. The patient was weaned and extubated in the operating room. Postoperative echocardiographic evaluation showed a trivial residue on ventricular septal defect and a 14 mmHg gradient on the pulmonary artery (Additional file 2: Fig. S2). The patient was transferred to the ward on the post-operative 2nd day and discharged on the 9th day. Thirteen months and eighteen months after the second operation, before and after her fifth dose, the patient’s Hammersmith score was 25 and 41, accordingly. In the patient’s recent follow-up at age of four, cardiac assessments were normal. In her physical and neurological examination, patient had a positive Gower’s sign with evident hypotonia but could walk unsupported, reported improvement in motor skills with continuing treatment and received a Hammersmith score of 64 (Additional file 3: Fig. S3).

## Discussion

Spinal muscular atrophy is one of the leading causes of infantile mortality. Reports of cardiac pathology co-existence are more frequent among the severely affected patients [3, 4]. Thus, supporting the notion that association with cardiac pathologies is related to prognosis [3]. Similar to our case, structural heart anomalies, mainly septal, and outflow tract defects are the most frequently expected pathologies [3]. Notably, a majority of the reported cases of structural cardiac pathologies are mostly in SMA type 1 patients [3, 4]. In patients with less severe forms of SMA, as in the case of our patient, congenital heart defects have rarely been reported [4].

To the best of our knowledge, there are no reported cases of successful surgical repair of heart defects in spinal muscular atrophy patients. Despite the patient’s asymptomatic neurologic condition, the unfavorable disease prognosis influenced our decision to initially perform a palliative procedure. With the introduction of disease-modifying medical treatments, spinal muscular atrophy is now associated with a better survival overall [1]. In addition, the considerably enhanced capacity for surgical correction of the heart has transformed the perspective, and surgical outcomes for many patients with hereditary syndromes are now much improved than they were previously [5]. Thus, for SMA patients with associated cardiac anomalies, a surgical treatment approach may be possible as presented in this case. Hereafter, in a patient with SMA, we would prefer a single-step total cardiac surgical correction approach rather than a two-step correction with an initial palliation.

The severe muscle weakness in SMA includes the respiratory muscles [3, 4, 7]. These patients are at increased risk of peri- and postoperative respiratory complications [7]. Prolonged intubation, difficult and failed intubations have been detailed in several case reports, unlike our experience with this patient [7]. There is currently minimal information on anesthetic management of SMA patients, and there are no standardized approaches for this population [7]. The uncomplicated post-operative recovery of our patient is a promising case for this disease group.

In a published clinical trial, Nusinersen-treated infants with spinal muscular atrophy had better survival and motor function than those in the control group [1]. Further, the results of the NURTURE study revealed that infants and children who started treatment with Nusinersen during the presymptomatic period attained motor milestones in timelines consistent with normal development [8]. As indicated by clinical and preclinical data, early treatment is important for optimal treatment efficacy: best outcomes are obtained when treatment is started before significant degeneration has occurred [1, 9]. With regards to our case, despite the late treatment onset of our patient, the progress in her motor milestones is promising as documented by her Hammersmith scores [6]. In addition, we made the decision regarding the second operation by virtue of the promising symptomatology and the favorable response the patient had with treatment. Hence, the disease-modifying drugs for SMA not only help improve the symptoms associated with the pathology but also enable for a more flexible management of the associated comorbidities. On the other hand, this is a costly treatment: according to the estimates from 2020, the price of Nusinersen is $776,000 in the first year and $388,000 per year for the subsequent years [10]. The cost of this treatment may prevent early therapeutic intervention. Although Nusinersen is widely approved for utilization in North America and Europe, cost coverage is variable especially in developing countries and regulations around treatment approval and coverage are in process [11]. Consequently, decisions regarding the use of Nusinersen are influenced by accessibility and cost [11]. Given the potential of this drug at improving functional survival, it is clear that a governmental policy is needed to ensure improved and early access to treatment. Performing a single-step correction instead of a two-step surgical correction with a palliative approach will improve the efficacy of resource utilization in the treatment of SMA patients in future cases.

## Conclusion

Cardiac pathologies in spinal muscular atrophy patients can be perceived as risky surgical corrections with suboptimal postoperative prognosis given the malign nature of the disease. Despite the poor prognosis of SMA, patients undergoing disease modifying treatment may be promising potential candidates for surgical cardiac correction.

## Supplementary Information

**Additional file 1: Fig. S1.** Postoperative echocardiographic imaging of the pulmonary band.

**Additional file 2: Fig. S2.** Postoperative echocardiographic imaging of ventricular septal defect closure.

**Additional file 3: Fig. 3.** Video of the presented patient in her daily life depicting her neurologic development.

## Data Availability

Not applicable.

## Abbreviations

SMA: Spinal muscular atrophy
SMN: Survival motor neuron

## References

[1] Finkel RS, Mercuri E, Darras BT, Connolly AM, Kuntz NL, Kirschner J (2017). Nusinersen versus sham control in infantile-onset spinal muscular atrophy. N Engl J Med.

[2] Lorson CL, Rindt H, Shababi M (2010). Spinal muscular atrophy: mechanisms and therapeutic strategies. Hum Mol Genet.

[3] Wijngaarde CA, Blank AC, Stam M, Wadman RI, van den Berg LH, van der Pol WL (2017). Cardiac pathology in spinal muscular atrophy: a systematic review. Orphanet J Rare Dis.

[4] Djordjevic SA, Milic-Rasic V, Brankovic V, Kosac A, Vukomanovic G, Topalovic M (2021). Cardiac findings in pediatric patients with spinal muscular atrophy types 2 and 3. Muscle Nerve.

[5] Formigari R, Michielon G, Digilio MC, Piacentini G, Carotti A, Giardini A (2009). Genetic syndromes and congenital heart defects: how is surgical management affected?. Eur J Cardiothorac Surg.

[6] Ramsey D, Scoto M, Mayhew A, Main M, Mazzone ES, Montes J (2017). Revised Hammersmith Scale for spinal muscular atrophy: a SMA specific clinical outcome assessment tool. PLoS ONE.

[7] Graham RJ, Athiraman U, Laubach AE, Sethna NF (2009). Anesthesia and perioperative medical management of children with spinal muscular atrophy. Paediatr Anaesth.

[8] De Vivo DC, Bertini E, Swoboda KJ, Hwu WL, Crawford TO, Finkel RS (2019). Nusinersen initiated in infants during the presymptomatic stage of spinal muscular atrophy: interim efficacy and safety results from the phase 2 NURTURE study. Neuromuscul Disord.

[9] Glascock J, Sampson J, Haidet-Phillips A, Connolly A, Darras B, Day J (2018). Treatment algorithm for infants diagnosed with spinal muscular atrophy through newborn screening. J Neuromuscul Dis.

[10] Dangouloff T, Botty C, Beaudart C, Servais L, Hiligsmann M (2021). Systematic literature review of the economic burden of spinal muscular atrophy and economic evaluations of treatments. Orphanet J Rare Dis.

[11] Michelson D, Ciafaloni E, Ashwal S, Lewis E, Narayanaswami P, Oskoui M (2018). Evidence in focus: Nusinersen use in spinal muscular atrophy: report of the guideline development, dissemination, and implementation subcommittee of the American Academy of Neurology. Neurology.

